# Caradiophyodidae, a New Family of Micro-Wasps (Hymenoptera: Platygastroidea) Based on the Description of *Caradiophyodus saradae* gen. et sp. nov. in Mid-Cretaceous Burmese Amber

**DOI:** 10.3390/life13081698

**Published:** 2023-08-07

**Authors:** George Poinar, Fernando E. Vega

**Affiliations:** 1 Department of Integrative Biology, Oregon State University, Corvallis, OR 97331, USA; 2 Independent Researcher, 14609 Pebblestone Dr., Silver Spring, MD 20905, USA; trainofstories@gmail.com

**Keywords:** coiled ovipositor, fossils, Myanmar, 15-segmented antennae

## Abstract

The female micro-wasp in mid-Cretaceous Burmese amber is described as a new genus and species in the extinct family Caradiophyodidae fam. nov. (Hymenoptera: Platygastroidea). Features of the specimen are its small body size (1.3 mm), no elbows, elongated, 15-segmented antennae, a deep cleft in the top of its head, a tarsal formula of 5-5-5, a reduced venation with a small pterostigma but no uncus in the forewing, no anal lobe in the hind wing, and a possible coiled ovipositor in the metasoma. Large unidentified expanded structures, considered to be possible seeds, plant secretions, or host eggs, are attached to each antenna.

## 1. Introduction

Fossils preserved in amber result from insects, leaves, flowers, and many other life forms coming in contact with resins from specific trees and eventually becoming entombed in the resin [1]. In Myanmar (Burmese) amber, the resins are produced by *Agathis* species (Araucariaceae) [1], and to date, over 2200 arthropod species have been identified in Myanmar amber [2].

Amber is the best medium for the preservation of micro-wasps with a body length under 5 mm since the morphological details of such wasps are rarely retained in sedimentary deposits. Burmese amber has yielded evidence of over 45 families of wasps, including many micro-wasps of the superfamily Platygastroidea whose lengths can be even less than 1.0 mm [3]. 

The Platygastroidea is a diverse group with some 260 genera and 6000 species, most of which are larval or egg-larval parasitoids of a wide selection of insect orders [4,5]. The female wasp described in this present study, with a body length of 1.3 mm, is assigned to the extinct family Caradiophyodidae fam. nov. based on various features listed below, especially the 15-segmented antennae [6].

A curious trait of this new Burmese amber specimen is the presence of an expanded structure attached to each antenna. Whether these attachments are plant seeds, host eggs, or secretions from its insect host could not be determined. This present paper describes the wasp and discusses some of its unusual morphological features.

## 2. Materials and Methods

The amber specimen originated from the Noije Bum Summit Site mine in the Hukawng Valley, located southwest of Maingkhwan in Kachin State (26°20′ N, 96°36′ E) in northern Myanmar. Based on paleontological evidence, the site was dated to the late Albian of the Early Cretaceous [7], placing the age at 97–110 Ma. A zircon U-Pb and trace element analyses of amber from different locations in Myanmar confirmed an age of around 100 Ma for amber from the Hukawng Valley as well as an age range of 72 Ma to 110 Ma for amber from other sites in northern and central Myanmar [8]. Observations and photographs were made with a Nikon SMZ-10 R stereoscopic microscope and Nikon Optiphot compound microscope with magnifications up to 800×. Helicon Focus Pro X64 was used to stack photos for better depth of field.

Terms used to describe basic structural characters are taken from Goulet and Huber [2], with additional features supplied by Chen et al. [5], Talamas et al. [6], and Mikó et al. [9]. The specimen is complete; however, the fore and mid legs are bent back on themselves, making features of the basal portions of the legs difficult to interpret. A male scale insect (Hemiptera: Coccomorpha) is the only syninclusion present. Nomenclatural acts introduced in this present work are registered in ZooBank (www.zoobank.org) under LSID urn:lsid:zoobank.org:pub:495DC246-9BF7-4276-82A0-680E38561125 (accessed on 24 July 2023).

## 3. Results

### Systematic Paleontology

Order: Hymenoptera Linnaeus, 1758.

Infraorder: Proctotrupomorpha Latreille, 1802.

Superfamily: Platygastroidea Haliday, 1833.

Family: Caradiophyodidae fam. nov. Diagnosis: for the only included genus.

LSID: urn:lsid:zoobank.org:pub:495DC246-9BF7-4276-82A0-680E38561125 (accessed on 24 July 2023).

**Diagnosis.** Antennae with 15 segments; flagellomeres mostly transverse; pedicel with a ventral flange, longer than scape; papillary sensilla on the surface of the apical antennomeres; dorsum of the head with distinct curved “horseshoe shaped” raised sclerite along inner margin of eyes; subocular sclerites positioned below eyes; head dorsum with a horizontal dorsal cleft between eyes and subocular sclerites; pronotum extending back to tegula; pronotal carina absent; fore wing with small pterostigma and submarginal vein reaching wing margin; uncus absent; telescoping ovipositor apparently present.

Genus: *Caradiophyodus* gen. nov. (Figure 1, Figure 2, Figure 3, Figure 4, Figure 5, Figure 6, Figure 7, Figure 8, Figure 9, Figure 10, Figure 11, Figure 12 and Figure 13).

Diagnosis: for the only included species.

LSID: urn:lsid:zoobank.org:act:418316E5-1BC0-4169-9582-4A2107F78B48 (accessed on 24 July 2023).

Etymology: “Cara” is from the Greek “Kara” = head, and “diaphyodus” is from the Greek “diaphye” = cleft or break, in regard to the cleft in the head of the fossil.

Type species: *Caradiophyodus saradae* gen. and sp. nov.

LSID: urn:lsid:zoobank.org:act:C9BD5866-D6E0-4278-B8A3-48C2DAE79CD1 (accessed on 24 July 2023).

**Figure 1:**
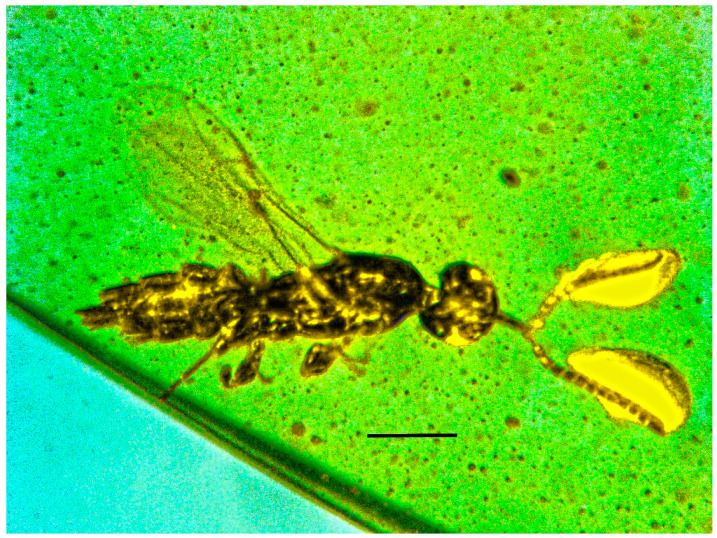
Lateral dorsal view of holotype of *Caradiophyodus saradae* gen. and sp. nov. in Burmese amber. Scale bar = 290 µm.

The specific epithet is in honor of Dr. Sarada Krishnan for her contributions to coffee genetic resources research. Dr. Krishnan is the Director of Programs at the Global Crop Diversity Trust in Bonn, Germany, and Executive Director of the International Women’s Coffee Alliance.

Holotype: Accession number B-Hy-21 deposited in the Poinar Amber Collection maintained at Oregon State University.

Type locality: Myanmar (Burma), state of Kachin, Noije bum 2001 Summit Site amber mine in the Hukawng Valley, SW of Maingkhwan (26°20′ N, 96°36′ E).

**Figure 2:**
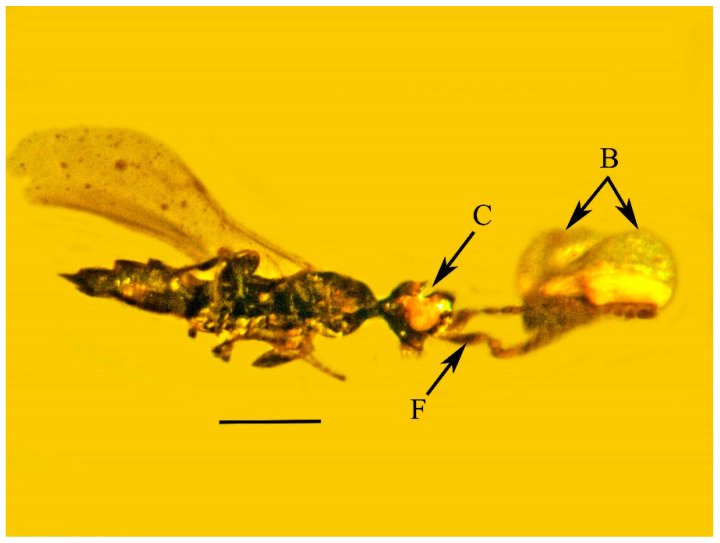
Lateral view of holotype of *Caradiophyodus saradae* gen. and sp. nov. in Burmese amber. C = cleft in head; F = flange on pedicel; B = expanded structures attached to antennae. Scale bar = 324 µm.

**Description:** Female. Body (length without antennae 1.3 mm) elongated, black, smooth, non-metallic, and nearly parallel-sided.

**Figure 3:**
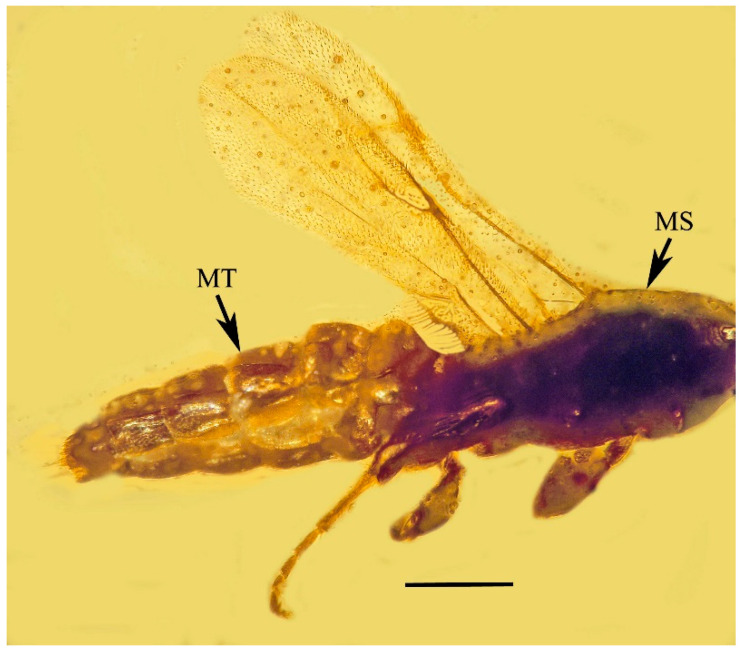
Lateral view of metasoma (MT) and mesosoma (MS) of holotype of *Caradiophyodus saradae* gen. and sp. nov. in Burmese amber. Scale bar = 150 µm.

**Figure 4:**
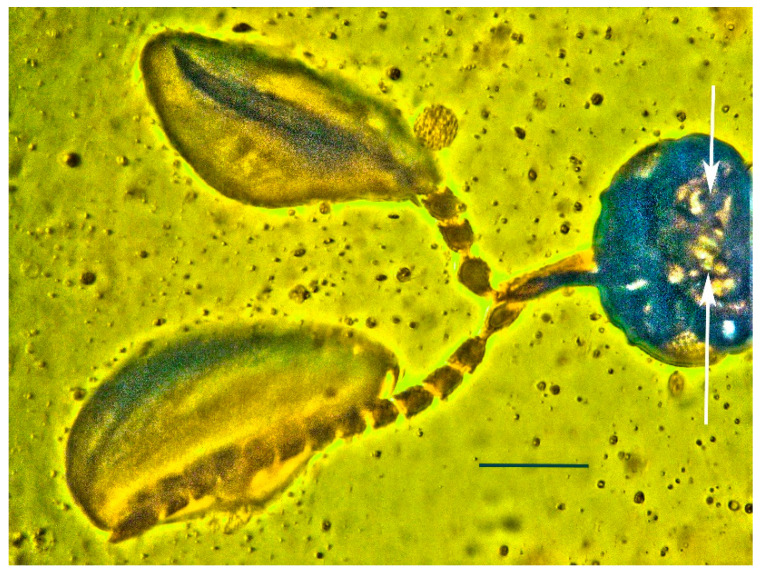
Head, antennae, and head cleft (between arrows) of holotype of *Caradiophyodus. saradae* gen. and sp. nov. in Burmese amber. Scale bar = 130 µm.

**Head:** Hypognathous; length, 210 µm; width, 277 µm; toruli adjacent; positioned just above oral cavity below level of ventral margin of eyes; much closer to each other than to eyes; length eye, 90 µm; ommatidia stalked; antennae 0.7 mm in length; not elbowed, with 15 segments; ring segments absent; scape short, length 36 µm, pedicel long, length 70 µm, with a ventral flange; first flagellomere fusiform (tapering at both ends), remaining flagellomeres transverse, gradually becoming larger toward apex and terminating in a 7- or 8-segmented clavis; dorsum of head with distinct curved, raised “horseshoe shaped” sclerite along inner margin of eyes, ending just before lateral ocelli, greatest diameter of sclerite, 160 µm; thickness of sclerite, 18 µm; dark subocular sclerite positioned below each eye; length suboptical sclerite, 92 µm; width suboptical sclerite, 53 µm; horizontal cleft extending across head between eyes and subocular sclerites.

**Mesosoma:** Black, smooth, lacking any metallic colors; length, 625 µm; pronotum extending back to tegulae; length pronotum 280 µm; pronotal carina absent; mesoscutum (length = 107 µm) transversely carinate; with faint ridges possibly incorporating the skaphion; notauli and petiole absent; mesoscutellum (length = 190 µm) squarish; carinate with faint transverse suture, lacking projection or process.

**Figure 5:**
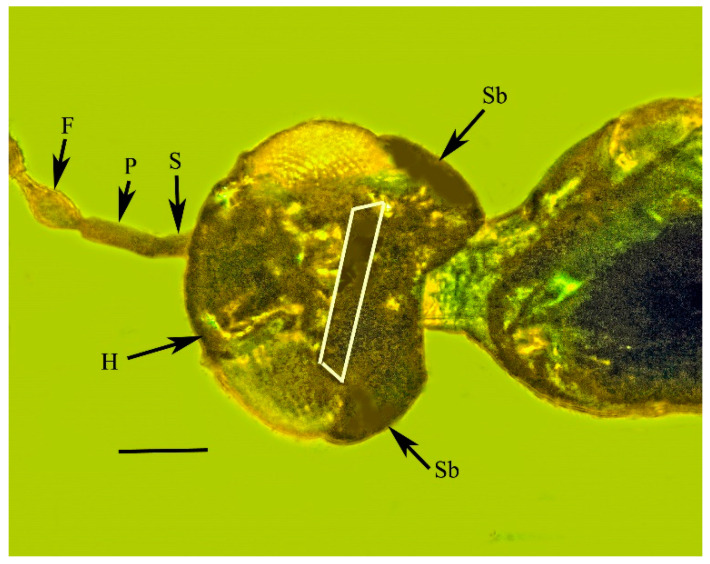
Head showing horseshoe sclerite (H); fusiform first flagellomere (F); pedicel (P); scape (S); subocular sclerites (Sb); and area of cleft (area within the white box) of holotype of *Caradiophyodus saradae* gen. and sp. nov. in Burmese amber. Scale bar = 97 µm.

**Legs:** Femora swollen; similar in size; length, 160 µm; tibia narrow; straight; length metatibia, 140 µm; tibial spur formula 1-1-2; tarsus 5-segmented, basal segment longest, segment lengths (1, 156 µm; 2, 60 µm; 3, 60 µm; 4, 43 µm; 5, 57 µm; pretarsus, 35 µm); tarsal claws paired, pointed; with wide base; aroleum positioned at right angles to the tarsus.

**Wings:** Forewing long (length, 850 µm; greatest width, 315 µm) extending almost to tip of the abdomen when directed backward; membrane and margins of both wings bearing setae; venation reduced; with no closed cells; distinct submarginal vein in fore wing meets wing margin at small pterostigma, then continues a short distance as a postmarginal vein; a straight stigmal vein extends down from the margin at roughly 45 degrees for a short distance; uncus absent; Rs vein incomplete; hind wing narrow (length, 500 µm, width, 60 µm); membrane covered with setae and bearing a short straight vein originating from near wing base; hamuli absent; anal lobe replaced by extended setae.

**Metasoma:** Sessile, with six subequal visible segments and partly exposed terminal 7 segment showing the tip of ovipositor and ovipositor sheaths. What appears to be a coil-like housing of the ovipositor is present.

**Figure 6:**
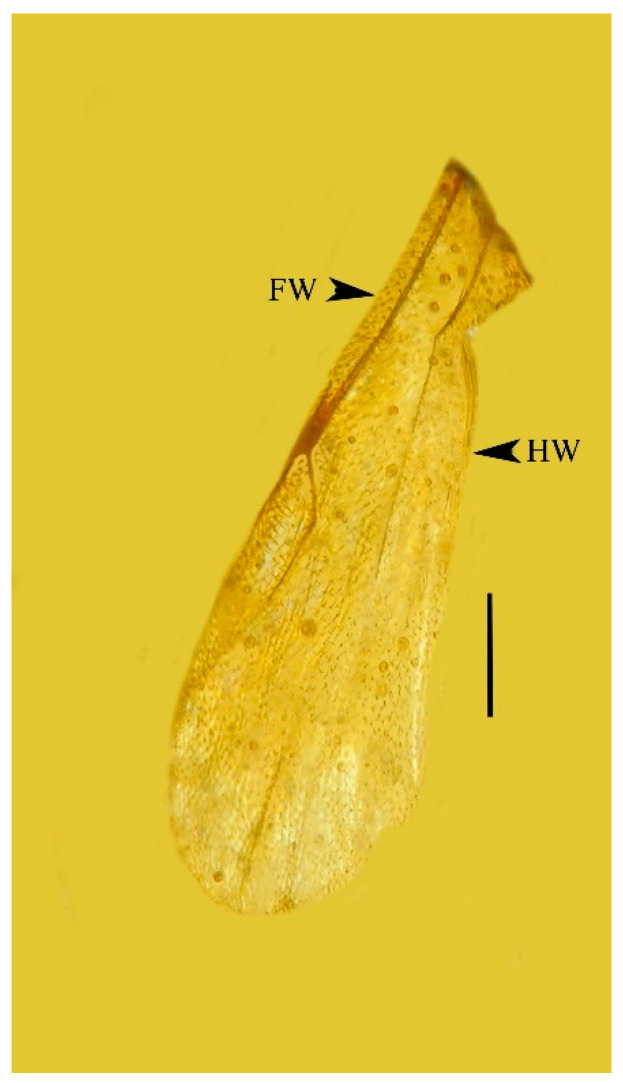
Forewing (FW) and hind wing (HW) of holotype of *Caradiophyodus saradae* gen. and sp. nov. in Burmese amber. Scale bar = 140 µm.

**Figure 7:**
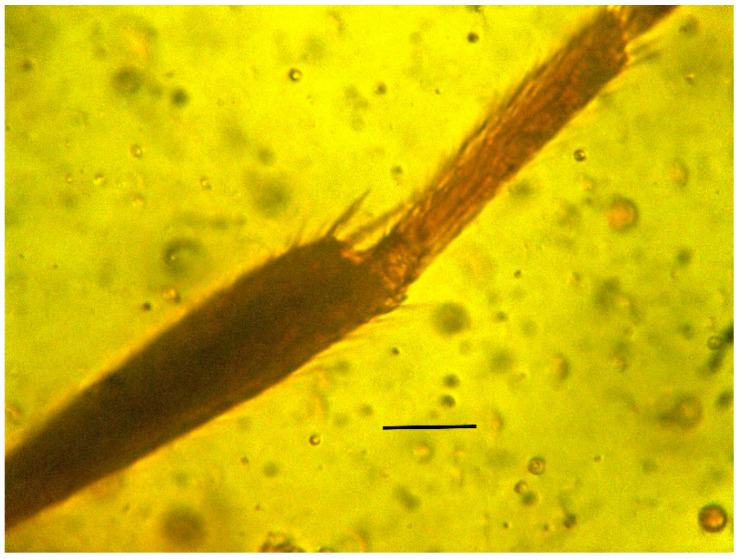
Metatibial spurs of holotype of *Caradiophyodus saradae* gen. and sp. nov. in Burmese amber. Scale bar = 40 µm.

**Comments:** Since the transition between the claval and funiclular antennomeres is so slight, it is difficult to determine if the club represents the terminal 6 or 7 segments [10]. The horizontal cleft extending across the head between eyes and subocular sclerites is considered a unique feature and could not be found in any other members of the Platygastroidea.

The 15-segmented antennae represent a primitive feature of the fossil and eliminate its placement in any of the previous families of the Platygastroidea [5] as well as in all species of the extinct Proterosceliopsidae, except for the Burmese amber *Proterosceliopsis plurima* Talamas, Shih, and Ren that also has 15 antennomeres [6]. The larger body size (3.9 mm versus 1.3 mm in *Caradiophyodus saradae* gen. and sp. nov.), nine clavomeres (versus 6 or 7 in *Caradiophyodus saradae* gen. and sp. nov.), extended metasoma, long pedicel, and long sixth tergite also separate the latter species from *Caradiophyodus saradae* gen. and sp. nov.

Aside from the above, members of the Proterosceliopsidae are characterized by a malarsulcus, a mesepimeral sulcus, a transepisternal line on the mesopleuron, and transverse furrows along the anterior margins of the tergites and sternites [2,6], which are not present on *Caradiophyodus saradae* gen. and sp. nov.

*Caradiophyodus saradae* gen. and sp. nov. also differ from the members of the Proterosceliopsidae in the following characteristics. The pronotal cervical sulcus of *Caradiophyodus saradae* gen. and sp. nov. is not furrowed and associated with glandular excretions as in members of the Proterosceliopsidae [6]. In addition, the wing of members of the Proterosceliopsidae has a bulla as well as evidence of a short, nebulous 1Rs vein and a 2Rs (=Rs) vein extending to the wing margin. In addition, members possess a nebulous Rs + M vein, nebulous to weakly sclerotized M and Cu veins, and a tibia spur formula of 1-2-2 [6], which differ from that of *Caradiophyodus saradae* gen. and sp. nov. The pedicel of the Burmese amber *Proterosceliopsis plurima* [6] is as long as the first three flagellomeres combined, while in *Caradiophyodus saradae* gen. and sp. nov., the pedicel length is slightly less than the first two flagellomeres. The abdomen is slender and petiolate in *P. plurima* [6] but is broad and sessile in *Caradiophyodus saradae* gen. and sp. nov. It is clear that the fossil does not belong to the Proterosceliopsidae.

Within the superfamily Platygastroidea, only the Burmese amber *Proterosceliopsis plurima* [6] and *Caradiophyodus saradae* gen. and sp. nov., possess 15 antennomeres, which is emphasized as a pleisomatic character by Talamas et al. [6]. Since *Caradiophyodus saradae* gen. and sp. nov. cannot be reliably assigned to any known extant or extinct family, it is placed in a new family.

The cleft in the head of *Caradiophyodus saradae* gen. and sp. nov. is considered a natural feature rather than a post-mortem crack. This is because there is no sign of torn or malformed adjacent head structures. The purpose of such a feature remains unknown.

**Figure 8:**
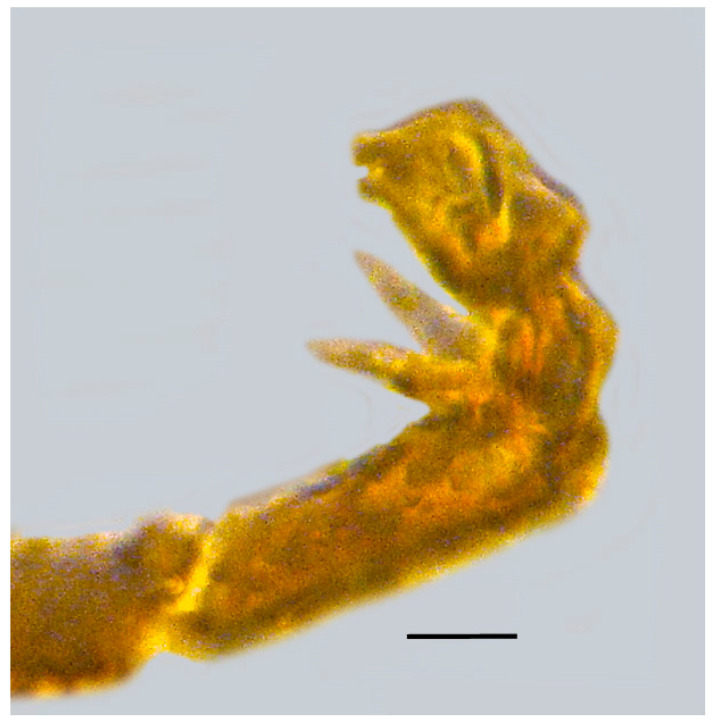
Foreleg pretarsus of holotype of *Caradiophyodus saradae* gen. and sp. nov. in Burmese amber. Scale bar = 12 µm.

## 4. Discussion

In some members of the families Platygastridae and Scelionidae, there is an internal adaptation for increasing the length of the ovipositor consisting of a coil-like housing of the ovipositor [2,5]. A structure of this nature occurs in the metasoma of *Caradiophyodus saradae* gen. and sp. nov. (Figure 10). The presence of a head cleft, a 7–8-segmented feeble club, short postmarginal vein, 15 segmented antennae, and other wing characters (Figure 2, Figure 4, Figure 5, Figure 6, Figure 12 and Figure 13) separates *Caradiophyodus saradae* gen. and sp. nov. from Baltic amber platygastrids, which includes species in the genera *Aneurobaeus*, *Archaeoscelio*, *Brachyscelio*, *Electroteleia*, *Proplatyscelio*, *Sembilanocera*, *Trachelopteron* and *Uroteleia* [11]. The Baltic amber *Sparaison simplicifrons* have the postmarginal vein extending to the tip of the radial cell, and the stigmal vein is curved [11,12,13]. The genus *Cobaloscelio* from Baltic amber has the mesopleuron and metapleuron completely fused; the frons has a well-developed median longitudinal carina, a short submarginal vein remote from the costal margin, and Rs as an arched nebulous vein [13]. The Baltic amber *Chromoteleia theobaldi* [14] has tri-dentate mandibles, and the postmarginal vein is much longer than that of *Caradiophyodus saradae* gen. and sp. nov.

**Figure 9:**
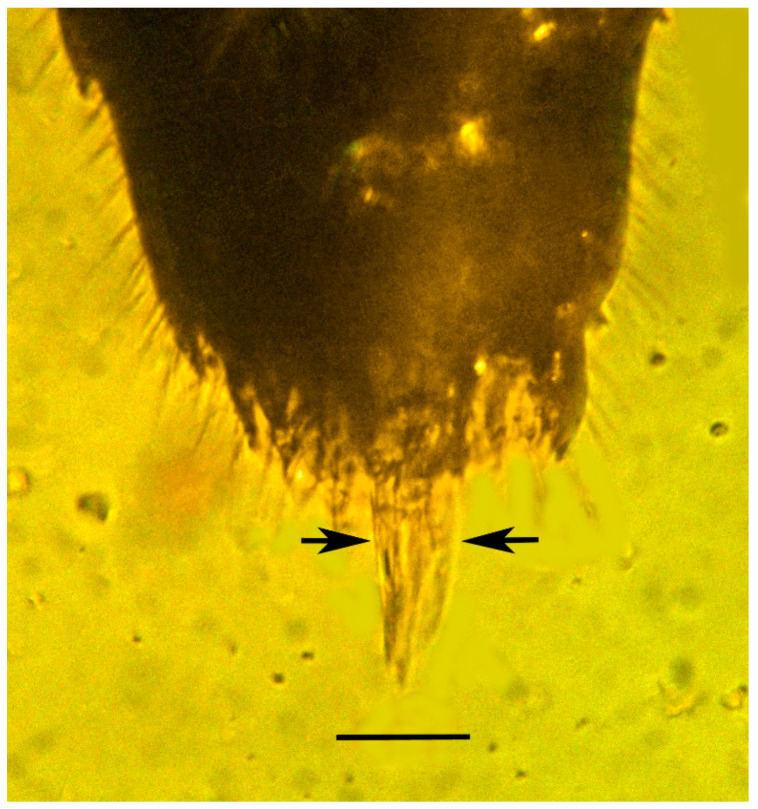
Terminalia of holotype of *Caradiophyodus saradae* gen. and sp. nov. in Burmese amber showing tip of ovipositor flanked by paired ovipositor sheaths (between arrows). Scale bar = 34 µm.

The Mexican amber *Palaeogryon muesebecki* [15] is only 0.6 mm in length, and the club is only 3-segmented. The genus *Moravoscelio* [16] in Eocene Moravian amber has reduced antennal segments 3–6 and a completely different forewing venation than *Caradiophyodus saradae* gen. and sp. nov. The genus *Galloscelio* [16] in French Eocene Oise amber has a 6-segmented club, and the venation in both fore and hind wings differ significantly from that of *Caradiophyodus saradae* gen. and sp. nov.

Three species of scelionids were described from Upper Cretaceous Canadian amber [17]. Of these, *Baryconus fulleri* [17] has the third tergite much longer than T1 and T2 combined, and the marginal vein is only half as long as the stigmal vein. *Baeomorpha dubitata* [17] has 9-segmented antennae inserted high on the face with a large compact club. *Proteroscelio antennalis* [17] has a flattened head and 14-segmented antennae. The Late Cretaceous *Cenomanoscelio pulcher* [18] from French amber has 11-segmented antennae, antennomeres 4–6 are reduced and transverse, and a large head with the eyes occupying most of the lateral surface. The Early Cretaceous Lebanese amber *Proteroscelio gravatus* [19] has a 14-segmented antenna and a wing venation that differs from that of *Caradiophyodus saradae* gen. and sp. nov. A list of Burmese amber micro-wasp genera with features that separate them from *Caradiophyodus saradae* gen. and sp. nov. is presented in Table 1.

Among known hosts of the Platygastroidea are members of the order Hemiptera [4,5]. The presence of a male coccoid adjacent to *Caradiophyodus saradae* gen. and sp. nov. suggests that the developmental host of the wasp were eggs or nymphs of a scale insect.

*Caradiophyodus saradae* gen. et sp. nov. has many attributes of encyrtids (Hymenoptera: Encyrtidae), especially species of the genus *Anagyrus* Howard, 1896 that have a similar body size, sessile abdomen, swollen pedicel, and reduced wing venation. The two genera differ in the number of antennomeres, with 15 in *Caradiophyodus* and 12 in *Anagyrus* spp. [20].

An expanded structure is attached to each antenna of *Caradiophyodus saradae* gen. and sp. nov. The length of these structures is 426 µm and 395 µm, respectively, and the greatest width is 192 µm and 220 µm, respectively (Figure 1, Figure 2, Figure 4, and Figure 11). While their identity remains unknown, they are definitely not attached to air bubbles. They have a definite surface pattern as well as an internal structure (Figure 11).

The great majority of *Anagyrus* species are endoparasites of mealybugs (Homoptera: Coccoidea: Pseudococcidae) [4,20]. Female *Anagyrus* spp. and related species that attack mealybugs derive food from honeydew or the body fluids of their hosts [4]. Some species feed on secretions from the puncture hole made after the wasp eggs are introduced [4]. Mealybug eggs are roughly the same shape and size as the unknown structures on the antennae of *Caradiophyodus* [21]. This provides a possible identification of the antennal structures attached to *Caradiaphyodus*: namely that they are host coccoid eggs that adhered to the flagellomeres after oviposition.

**Table 1:** List of Burmese amber micro-wasp genera with features that separate them from *Caradiophyodus saradae* gen. and sp. nov.

Wasp Genus	Family	Features	Reference
*Baeomorpha*	Rotoitidae	13 antennomeres; 6-segmented clava; small body (ca. 645 µm); basal vein present	[22]
*Burminata*	Diversinitidae	13 antennomeres; 3-segmented clava; basal vein present; mulriporous plate sensilla	[23]
*Cascoscelio*	Scelionidae	12 antennomeres; ocelli on tubercle; 5-segmented clava; uncus present; hind wing with submarginal vein	[24]
*Christophus*	Diapriidae	14 antennomeres; radial cell closed; forewing oval-shaped; M + Cu vein present	[25]
*Diversinitus*	Diversinitidae	13 antennomeres; 3-segmented clava; basal vein present; mulriporous plate sensilla	[23]
*Gallorama*	Mymarommatidae	wing fringe; 2-segmented petiole; 3-segmented clavus	[26]
*Glabiala*	Diversinitidae	13 antennomeres; 3-segmented clava; basal vein present; mulriporous plate sensilla	[23]
*Mintara*	Diapriidae	14 antennomeres; antennae inserted on shelf; radial cell closed; M + Cu vein present	[25]
*Proterosceliopsus*	Proterosceliopsidae	bulla present; short, nebulous 1Rs vein; nebulous Rs + M vein; Rs vein extending to the wing margin; tibial spur formula of 1-2-2	[6]
*Protobelyta*	Diapriidae	antennae on distinct shelf; forewing with radial cell; hind wing with basal cell; petiole present	[27]

**Figure 10:**
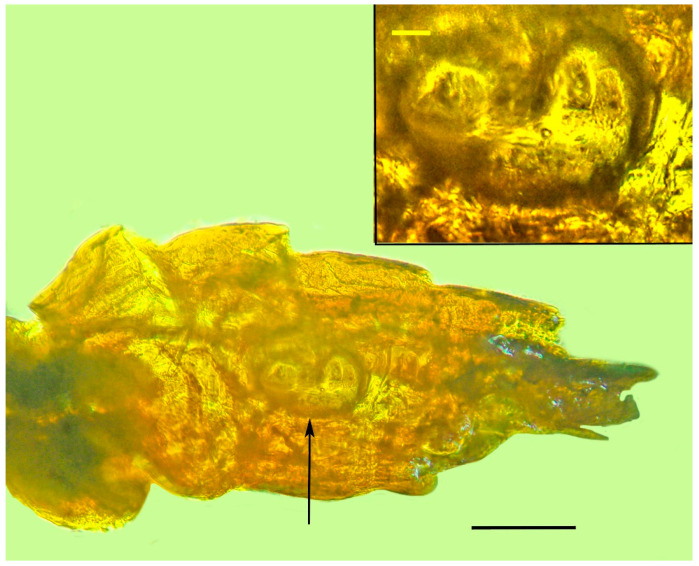
Lateral abdominal sclerites showing a possible coiled ovipositor (arrow) of holotype of *Caradiophyodus saradae* gen. and sp. nov. in Burmese amber. Scale bar = 14 µm. Insert shows structure with possible developing eggs. Scale bar = 4.1 µm.

**Figure 11:**
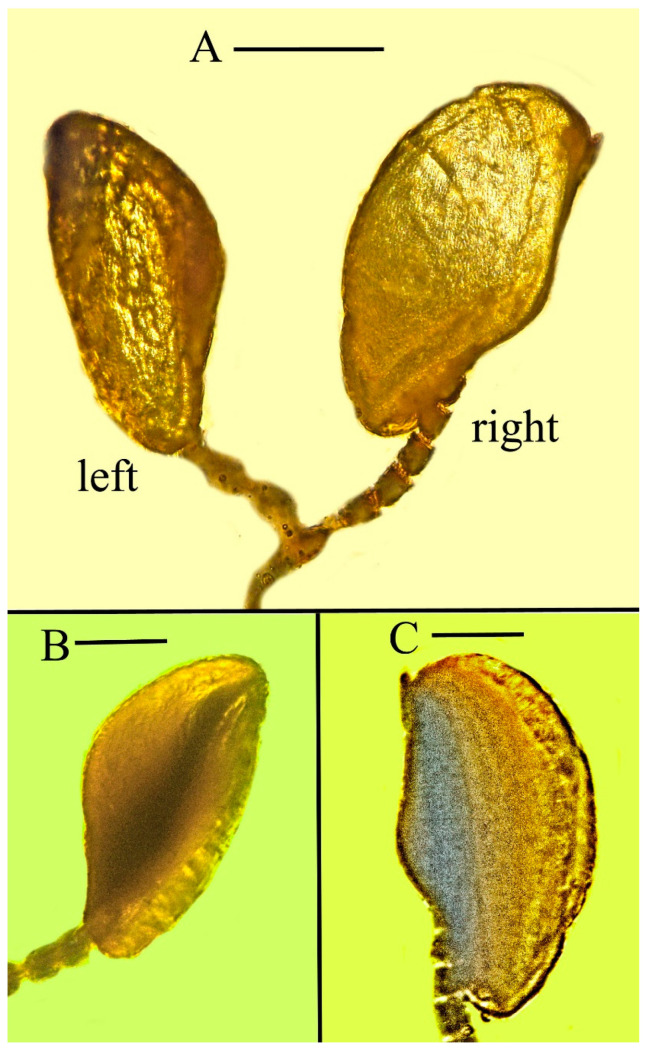
Expanded structures attached to the antennae of holotype of *Caradiophyodus saradae* gen. and sp. nov. in Burmese amber. (**A**) Structures on left and right antennae. Scale bar = 180 µm. (**B**) Opposite side of structure on left antenna. Scale bar = 120 µm. (**C**) Opposite side of structure on right antenna. Scale bar = 100 µm.

**Figure 12:**
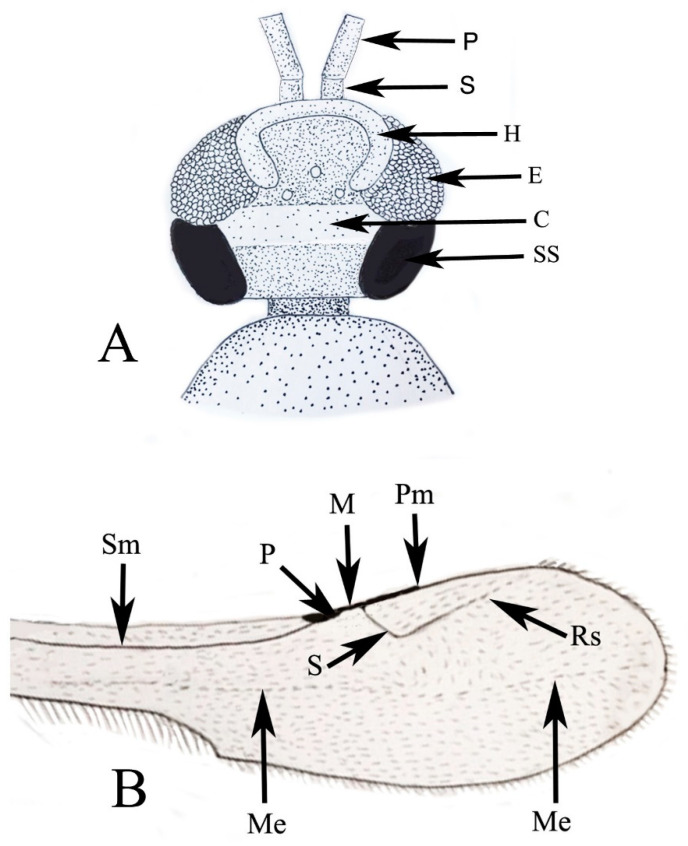
Basic features presented in stylized drawings of holotype of *Caradiophyodus saradae* gen. and sp. nov. in Burmese amber. (**A**) Head. C = cleft; E = eye; H = horseshoe sclerite; P = pedicel; S = scape; SS = subocular sclerite. (**B**) Forewing venation. M = marginal vein; Me = nebulous medial vein; P = pterostigma; Pm = limit of postmarginal vein; Rs = Rs vein; S = stigmal vein; Sm = nebulous submarginal vein. Figures not drawn to scale.

**Figure 13:**
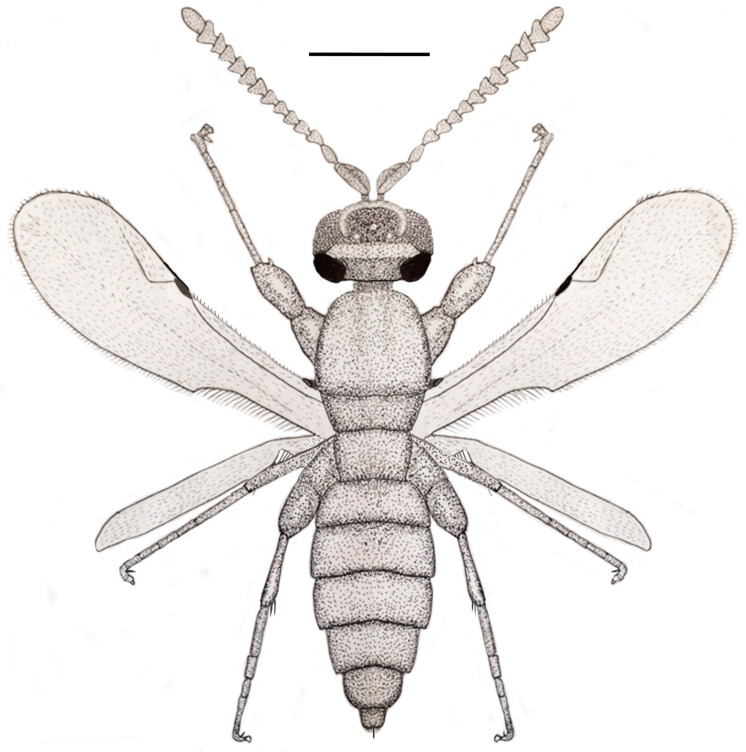
A reconstruction of the holotype of *Caradiophyodus saradae* gen. and sp. nov. in Burmese amber showing basic features. Scale bar = 0.3 mm.

## Data Availability

Not applicable.
